# The prevalence of alcohol use disorders among people living with HIV/AIDS: a systematic review and meta-analysis

**DOI:** 10.1186/s13011-019-0240-3

**Published:** 2019-11-14

**Authors:** Bereket Duko, Mohammed Ayalew, Getinet Ayano

**Affiliations:** 1 Faculty of Health Sciences, College of Medicine and Health Sciences, Hawassa University, Hawassa, Ethiopia; 2 Research and Training Department, Amanuel Mental Specialized Hospital, Addis Ababa, Ethiopia

**Keywords:** Prevalence, Alcohol use disorder, HIV/AIDS, Systematic review, Meta-analysis

## Abstract

**Background:**

Alcohol use disorder (AUD) is common among people living with HIV/AIDS (PLWHA) and associated with a greater risk of poor medication adherence, unsafe sexual behaviors as well as poor quality of life. To our knowledge, there is no previous systematic review and meta-analysis that reported the pooled prevalence estimate of AUD among PLWHA. Therefore, this review aimed to systematically review the available studies on the prevalence of AUD among PLWHA and forward possible recommendations for future clinical practice and research.

**Methods:**

PubMed, EMBASE, Psych INFO and SCOPUS databases were searched to identify the relevant studies. We have also scanned the reference lists of the eligible studies to supplement our electronic search. We used the Comprehensive Meta-Analysis software versions 3.0 to conduct a meta-analysis. Subgroup and sensitivity analysis were performed and Cochran’s Q- and the I^2^- test were employed to see the heterogeneity. The presence of publication bias was explored by utilizing Egger’s test and visual inspection of the symmetry in funnel plots.

**Results:**

A total of 25 studies with 25,154 participants across developed and developing countries were included in the final analysis. Our meta-analysis revealed that the pooled prevalence estimate of AUD among PLWHA was found to be 29.80% (95% CI; 24.10–35.76). The prevalence of AUD was higher in males (26.90%) than female (13.37%) HIV patients. In this study, the pooled prevalence of AUD was considerably higher (31.52%) when measured by Alcohol Use Disorders Identification Test (AUDIT) as compared to Composite International Diagnostic Interview (CIDI) (13.51%). In addition, the pooled prevalence of AUD was higher in the developed countries (42.09%) while lower for developing countries (24.52%).

**Conclusion:**

In the current study, the pooled prevalence estimates of AUD among PLWHA was considerably high (29.80%). Screening and appropriate management of AUD among PLWHA are recommended.

## Background

Human Immunodeficiency Virus (HIV) infection is a major public health concern that claimed the life of 770,000 people in 2018 [1]**.** Globally, 37.9 million people were living with HIV at the end of 2018 and 1.7 million people becoming newly infected with HIV in 2018 [1]. A significant number of HIV infected individuals suffer from substance use disorders [2].

The World Health Organization (WHO) defines substance use disorder as the harmful or hazardous use of any psychoactive substances, including alcohol and illicit drugs [3]. Kaplan & Sadock’s Synopsis of Psychiatry refers “alcohol use disorder (AUD) as the continuous use of alcohol despite evidence of harm and repeated attempts to cut down the use” [4]. Alcohol use disorder results in short and long term impacts on the physical, mental and socio-economic aspects of individual life [5]. Further, AUD contributes nearly 4% of the global burden of disease [6].

Alcohol use disorder is commonly left undiagnosed among people living with HIV/AIDS (PLWHA), which could potentially lead individuals to engage in a number of high-risk activities such as involvement in unsafe sexual engagement that might enhance the spread of HIV [7, 8] as well as compromise the antiretroviral therapy (ART) response [9].

A study conducted to assess the prevalence of non-HIV cancer risk factors in persons living with HIV/AIDS reported PLWHA are two to four times more likely to use alcohol than the general population [10]. Other similar studies also reported that 40 to 50% of individuals living with HIV/AIDS have a history of alcohol abuse or dependence [11, 12].

There are different rates of prevalence of alcohol use disorder among PLWHA across developed and developing countries [9–13]. For example, a cross-sectional study conducted in Nigeria to assess the prevalence of AUD among PLWHA revealed 39.4% [14] whereas, a prospective cohort study from the United States of America reported 12% prevalence [15].

Alcohol use disorder has a significant impact on PWLHA. For instance, AUD can interfere with the immune system of the body [14], predispose individuals to bacterial infections like tuberculosis [16], may result in liver damage**,** and alter the metabolism of antiretroviral drugs [17]. AUD is also associated with harmful behavior, such as tobacco smoking, illicit drug use and unsafe sexual practices [18].

The findings from the recent studies revealed AUD among PLWHA was linked with more serious cognitive problems such as reduced response time, executive functioning and verbal or visuospatial learning and memory [19, 20]. Furthermore, PLWHA with AUD have a poor health-related quality of life (QOL) which can put negative influence on the physical and emotional well-being of the individuals [20–23].

Even though a wide range of studies showed AUD as a significant public health importance, there is no systematic review and meta-analysis conducted to assess the prevalence of AUD among PLWHA. Therefore, this systematic review and meta-analysis aimed to summarize the existing evidence on the prevalence of AUD among PLWHA and to formulate possible suggestions for future clinical practice and research community.

## Methods/design

### Study design and search process

We performed an extensive search of literature as suggested by the guideline of reporting systematic review and meta-analysis (PRISMA) [24]. We have reviewed published literature conducted on the prevalence of AUD among PLWHA. The systematic literature search was conducted using electronic databases such as PubMed, EMBASE, Psych INFO, and SCOPUS. We performed our electronic search in PubMed using the following MeSH (Medical Subject Headings) terms: *“(alcohol use OR alcohol use disorder OR hazardous drinking OR alcohol abuse OR alcohol dependence)) AND (prevalence OR magnitude OR epidemiology)) AND (HIV OR human immune deficiency virus OR AIDS OR acquired immune deficiency syndrome)”.* The other databases such as EMBASE and SCOPUS were also comprehensively searched by applying the search terms used in PubMed for each database. In addition, we have manually searched the reference lists of eligible articles.

### Eligibility criteria

In this systematic review and meta-analysis, appraisal of the identified studies was done using their title and abstract before the retrieval of full-text articles for further screening by the two reviewers (BD and GA). We adhered a predefined inclusion and exclusion criteria to screen the retrieved full articles and any discrepancies were solved by a discussion with a third reviewer (MA). We have included the studies in the review using the following inclusion criteria: First, cross-sectional and other observational studies; Second, conducted among PLWHA; Third, studies that were published in the English language; finally, studies that determined the prevalence of AUD among PLWHA. Therefore, we excluded duplicate studies, commentaries, reviews, letters, editorials, and short communications as they did not satisfy the eligibility criteria.

#### Methods for data extraction and quality assessment

Two reviewers (BD and MA) conducted the data extraction from the relevant articles. The following information was extracted: the name of the first author, the year of publication, study setting, and design, sample size, prevalence, and tools used to estimate the magnitude of AUD, and the reported magnitude by gender of the participants (See Table 1). Disagreements raised during data extraction were solved by discussion and consensus. A modified and adapted version of the Newcastle-Ottawa Scale (NOS) [47, 48] was used to assess the quality of included studies in the meta-analysis (See Additional file 1). The data collection tool used to measure AUD, sample size, statistical quality, sample representativeness and comparability between participants were the domains of the NOS scale to assess the quality of the eligible articles.

**Table 1:** Characteristic of studies included in the systematic review and meta-analysis (Data extraction)

First author name, year	Country	Study design	Year of data collection	Sample size	Mean/median age of study participants (+ SD)	AUD measured by	No. of male with AUD	No. of female with AUD	Prevalence of AUD (%)
Silverberg et al., 2013 [25]	US	RCT	2013–15	614	49.4	AUDIT	NA	NA	48
Segni et al., 2017 [7]	Ethiopia	Cross-sectional	2016	418	NA	AUDIT	101	14	24.7
Silva et al., 2017 [26]	Brazil	Cross-sectional	2012–3	343	42.2 (11.1)	AUDIT	71	27	28.6
Crane et al., 2017 [27]	US	Longitudinal study	2013–2015	8567	NA	AUDIT	1983	287	27
Nouaman et al., 2018 [28]	Four African countries	Cross-sectional	2013–4	1824	39	AUDIT	256	136	38.4
Pokhrela et al., 2018 [29]	Nepal	Cross-sectional	2015	682	36.3 (8.2)	AUDIT	124	51	25.7
Egbe et al., 2017 [30]	Nigeria	Cross-sectional	2015	1187	39.3 (9.1)	CIDI	17	9	14
Rosmary, 2015 [31]	Kenya	Cross-sectional	2015	178	NA	AUDIT	27	9	33
Kibera et al., 2017 [32]	Kenya	Cross-sectional	2014	272	NA	AUDIT	33	5	14
Goar et al., 2011 [13]	Nigeria	Cross-sectional	NA	160	35.6 (8.66)	AUDIT	35	28	39.4
Mayston et al., 2015 [33]	India	Cross-sectional	2010	1934	35	AUDIT	NA	NA	12.8
Zelalem et al., 2018 [34]	Ethiopia	Cross-sectional	2017	358	NA	AUDIT	NA	NA	30.2
Oliveira et al.,2015 [35]	Brazil	Cross-sectional	NA	108	42.7 (9.4)	AUDIT	5	8	12
Soboka et al., 2014 [36]	Ethiopia	Cross-sectional	2012	401	35.5 (9.78)	AUDIT	63	64	32.6
Orwat et al., 2011 [14]	US	Cohort	2006	369	42.7	CIDI	NA	NA	12
Parsons et al.,2014 [37]	US	Cross-sectional	2013	557	55.0 (4.4)	AUDIT	NA	NA	74.3
Simon et al., 2014 [38]	Brazil	Cross-sectional	2008–9	580	40.6 (10.8)	AUDIT	77	46	21.2
Medley et al., 2014 [39]	SSA	RCT	2009–10	3538	37.2 (8.4)	AUDIT	108	76	20
Scott-Sheldon et al., 2014 [40]	South Africa	RCT	2008–10	763	30	AUDIT	NA	NA	62
Jolley et al., 2016 [41]	US	Cohort	2007–11	196	22	AUDIT	NA	NA	76
Idrisov et al., 2017 [42]	Russia	RCT	2012–14	249	44 (9)	AUDIT	NA	NA	59
Bultum et al., 2018 [43]	Ethiopia	Cross-sectional	2015	527	34.3 (4.8)	AUDIT	49	26	14.2
Wandera et al., 2015 [44]	Uganda	Cross-sectional	2014	725	36.54 (9.49)	AUDIT	112	127	33
Farley et al., 2010 [45]	Nigeria	Cross-sectional	2007	399	NA	AUDIT	48	28	12
Duko et al., 2019 [46]	Ethiopia	Cross-sectional	2019	195	29.88 (10.89)	AUDIT	42	20	29.9

#### Data synthesis and analysis

We used a Comprehensive Meta-Analysis software version 3.0 to conduct a meta-analysis. The random effect model for meta-analysis was used to measure the overall pooled prevalence of AUD among PLWHA**.** The magnitude of statistical heterogeneity between the eligible articles was measured by using Q statistic and the I^2^ statistics [49] and values of 25, 50 and 75% were used to represent low, medium and high quality respectively [50]. Subgroup and sensitivity analysis was conducted to determine the potential source bias. The instruments used to assess the presence or absence of AUD, the location of the studies as well as the quality of the included studies were used as a moderator to assess the sensitivity as well as subgroup analysis. The funnel plot and Egger’s regression tests were used to assess potential publication bias [51].

## Results

### Identification of studies

**Fig. 1:**
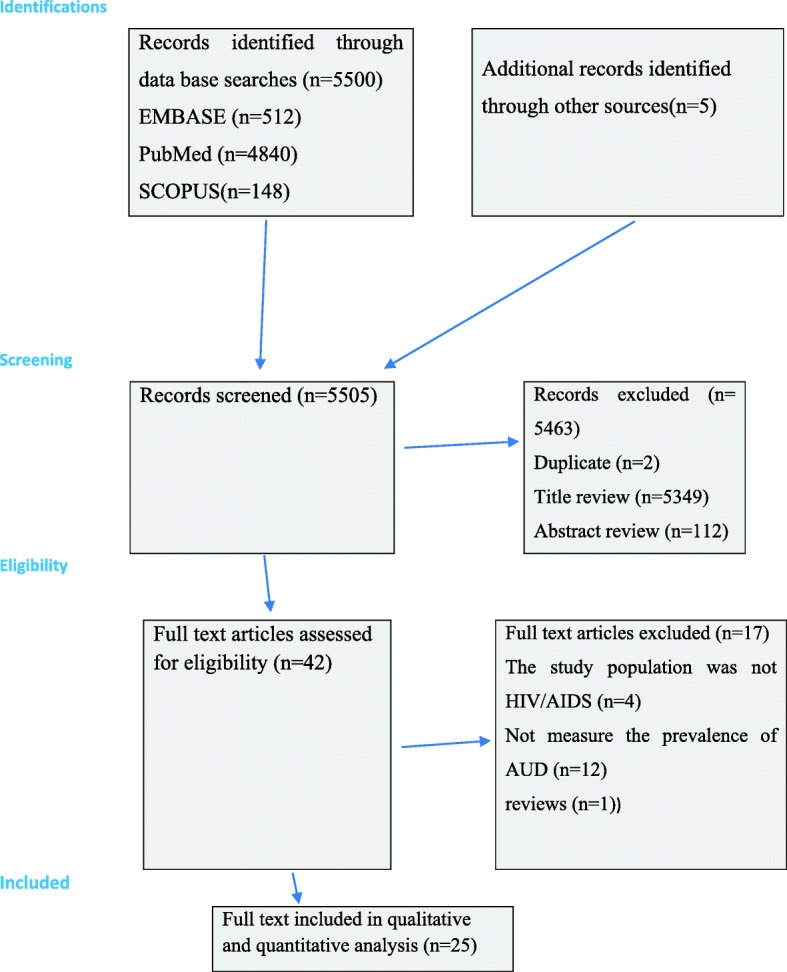
Shows the PRISMA flowchart of systematic review search

Our electronic database, as well as additional manual search, resulted in a total of 5505 documents. After a thorough screening, a full-text of 42 articles were retrieved for further appraisal and 17 of these were excluded (see Fig. 1).

### Characteristics of included studies

In the current systematic review and meta-analysis, a total of 25 articles conducted in developing and developed countries including 25,154 participants were included in the final meta-analysis. The characteristics of the included studies was illustrated in Table 1. The studies included in this review were published between 2006 and 2019, with the sample size ranging between 108 participants in Brazil and 8567 participants in the USA. Among the 25 studies, five were from the USA, three from Brazil, one from Russia, one from India, one from Nepal and 11 from African countries. AUD among PLWHA was predominantly measured using AUDIT [52]. Thus, the AUDIT was used in 23 studies while the CIDI was used in only 2 studies.

### Quality of included studies

Among the included articles, 19 articles were high quality (NOS score 8 and above), 5 articles were moderate quality (NOS score between 6 and 7 inclusive) and 1 article was low-quality studies (NOS score less than or equal to 5) (See Additional file 2).

### The prevalence of AUD among PLWHA (meta-analysis)

**Fig. 2:**
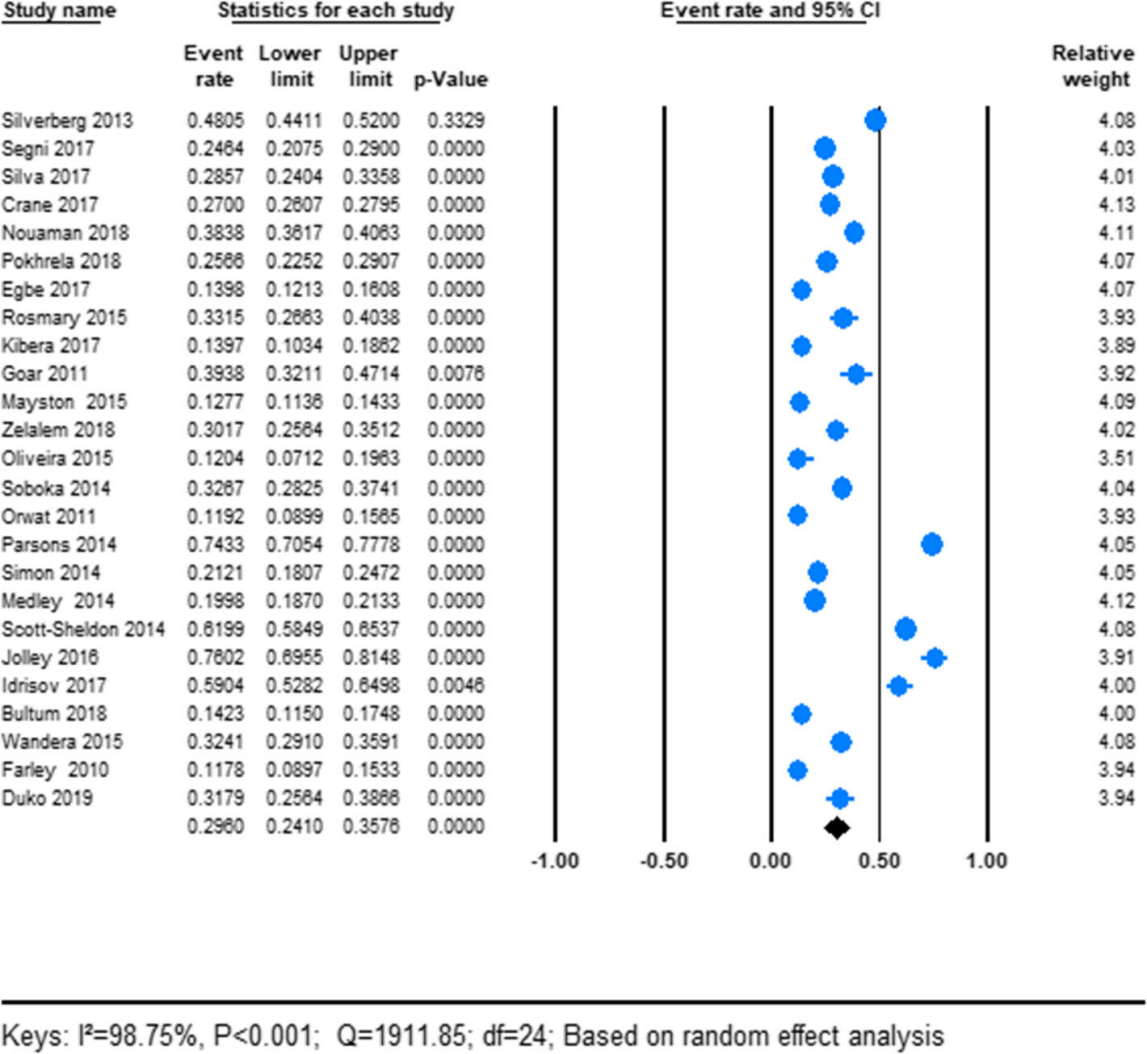
Shows the pooled prevalence of AUD among HIV patients: meta-analysis

In this review, twenty-five articles that reported the prevalence of AUD among PLWHA were included in the final analysis (Table 1). The pooled prevalence estimate of AUD among PLWHA was found to be 29.80% (95% CI; 24.10–35.76). We used a random-effect model due to the reported heterogeneity. We found an apparent heterogeneity for this analysis (*I*^2^ = 98.75%; *p* < 0.001) (See Fig. 2).

## Subgroup and sensitive analysis

The available epidemiologic evidence was diverse by the location of the study (the origin of the study), the instrument used to estimate AUD, the gender of participants as well as the methodologic quality.

### The prevalence of AUD among PLWHA by developed and developing countries

In our subgroup analysis of developing and developed countries as a moderator, we found a significantly higher prevalence of AUD in developed countries 42.09% (95% CI 27.29–58.47) while comparably lower prevalence in the developing countries 24.52% (95% CI 19.66–30.14). The variation between the countries was statistically significant (*P* < 0.001) (See Table 2).

**Table 2:** Sensitivity analysis of all studies based on study quality, tools used, gender and status of the country where the study is conducted

Subgroups	Studies, n	Prevalence (%)	95%CI	Heterogeneity within the study *(I*^*2*^*, Value)*		Heterogeneity between groups (*P value*)
				I^2^ (%)	*P* value	
Country						
Developed	8	42.09	27.29–58.47	99.38	<0.001	0.028
Developing	17	24.52	19.66–30.14	97.34	<0.001	
Tools used						
AUDIT	23	31.52	25.66–38.02	98.75	<0.001	<0.001
CIDI	2	13.51	11.86–15.34	2.13	0.312	
Gender						
Male	15	26.90	19.72–35.53	97.92	<0.001	
Female	15	13.37	8.79–19.80	97.03	<0.001	<0.001
Quality of studies						0.598
High	19	28.50	22.42–35.49	98.89	<0.001	
Moderate and poor	6	33.19	18.97–51.33	98.54	<0.001	

### Subgroup analysis of the prevalence of AUD by the instrument used

We further performed a subgroup analysis using the type of instrument used to assess AUD as a moderator. The pooled prevalence of AUD was considerably higher when measured by AUDIT 31.52% (95%CI; 25.66–38.02) as compared to CIDI 13.51% (95%CI; 11.86–15.34%). The heterogeneity was significant for the studies performed by AUDIT (*I*^2^ = 98.75%, *p* < 0.0001), but not for CIDI (*I*^2^ = 2.13%, 0 = 0.312) (See Table 2).

### Subgroup analysis of the prevalence of AUD among HIV patients by gender of participants

In this review, 15 studies reported the prevalence of AUD in male and female participants. Analysis of those previous studies which reported the prevalence of AUD by males and females revealed the higher prevalence in male 26.90% (95% CI, 19.72–35.53) than females 13.37% (95% CI, 8.79–19.80). A significant heterogeneity was found in both males (*I*^2^ = 97.92; *p* < 0.001) and females (*I*^2^ = 97.03; *p* < 0.001) (see Table 2).

### Subgroup analysis of the prevalence of AUD among HIV patients by the quality of studies

Finally, we also performed the sensitivity analysis based on the quality of the included studies. The prevalence AUD for high quality was 28.50% and it was 33.19% for moderate and low-quality studies, even though the difference was not statistically significant (*P* = 0.586) (see Table 2).

### Publication bias

**Fig. 3:**
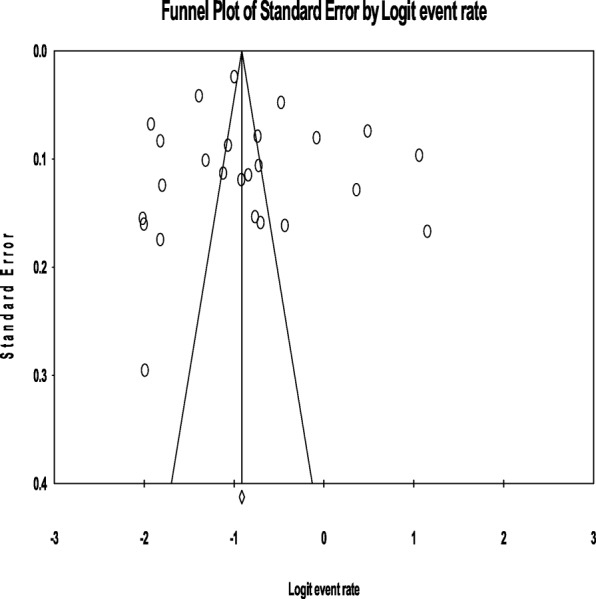
Shows the funnel plot of publication bias of the included studies

For the overall meta-analysis of the prevalence of AUD among PLWHA, the funnel plot was symmetric and Egger’s regression tests provided no evidence of potential publication bias (B = 1.69, SE = 3.38, *P* = 0.621) (See Fig. 3).

## Discussion

To our knowledge, this is the first systematic review and meta-analysis that explored the prevalence of AUD among PLWHA. The result of the pooled meta-analysis showed that the magnitude of AUD was remarkably high among PLWHA (29.80%). This finding indicates AUD is a significant public health issue among PLWHA.

In the present systematic review and meta-analysis, the available epidemiologic evidence was diverse by the location of the study (the origin of the study), the instrument used to estimate AUD, as well as the methodologic quality. Most of the studies used AUDIT while only 2 studies used CIDI to measure AUD. The majority of the included studies were of high quality (*n* = 19).

In this review, the pooled prevalence estimate of AUD among PLWHA (29.80%) was higher than the reported prevalence of AUD among the general population of the globe. For example, the studies conducted to assess the prevalence of AUD among the general population in the USA, Europe, and Australia reported 13.9, 11.1 and 11.8% respectively [53–55]. This variation might be due to people with the terminal illness such as HIV, may use alcohol as a coping mechanism for a way of dealing with psychological distress resulted due to the severity of the HIV illness and antiretroviral drugs side-effects [54, 55].

Contrarily, the pooled prevalence of AUD in our meta-analysis was lower than the prevalence of AUD among patients with schizophrenia, major depressive disorder and personality disorder which were 33.7, 28, and 50–70% respectively [56]. This may be due to the triggering effect of mental disorders. Thus, having mental disorders could result in alcohol use disorder in this group of population.

In our subgroup analysis, the pooled prevalence of alcohol use disorder was higher in men (26.90%) than women (13.37%). The difference in the prevalence of AUD among men and women may be due to the difference in the neurochemistry of the brain in the respective genders. For instance, a study conducted in the USA reported that regardless of the same level of alcohol drinking, a male has a higher dopamine release than a female [57]. The researchers of this study believe, the ability of alcohol to stimulate the release of dopamine may potentially contribute to its rewarding effect and this may be linked with a higher prevalence of AUD in males. Further, the difference in prevalence might be due to the variations in socio-cultural aspects of person’s life, for example, some cultures might not allow women to drink alcohol and also other environmental factors may play a role in this variation [58, 59].

The subgroup analysis using the instruments used to estimate AUD revealed a significant difference in the prevalence of AUD among PLWHA. The pooled prevalence of AUD was higher in studies conducted using AUDIT (31.50%) than CIDI (13.51%). The difference in instruments may contribute to the variation in the prevalence of AUD. For instance, AUDIT is a screening tool with aim of detecting early or risk factors of alcohol use disorder in a large number of asymptomatic whereas CIDI is a diagnostic tool which potentially depicts the presence or absence of AUD in symptomatic individuals to establish the diagnosis and arrange for clinical management [59].

Furthermore, the pooled prevalence estimates of AUD among PLWHIVA in developed countries (42.52%) was significantly higher than the pooled prevalence estimates of the developing countries (24.52%). This finding is in line with the other study conducted on alcohol abuse that revealed the prevalence of alcohol use in developed and developing nations which were 29.3 and 16.2% respectively [60]. In addition to this, the variation in the socio-economic status of the countries, cultural differences, the accessibility and availability of alcoholic beverages, variation in the number of studies in developed countries and the study setting capability of investigation might contribute to the difference in the prevalence of alcohol use among PLWHA.

### Strength and limitations

The strengths are: (1) We employed our search based on a predefined search strategy designed to minimize reviewer’s bias; (2) data extraction and quality appraisal were done by two independent authors; (3) Execution of the sensitivity and subgroup analysis, countries of the study, the gender of the participants and the AUD assessment tool used. However, the review has the following limitations. The small number of articles that were included in the subgroup analysis could decrease the accuracy of the estimate. Furthermore, the AUDIT questionnaire has high sensitivity (94.1%). This could overestimate the prevalence of AUD in the studies that used AUDIT to assess AUD. In addition, few studies (two) used CIDI to measure AUD. This could also limit the sub-group analysis when using tools of AUD as a moderator. In addition, the review included articles only published in the English language. This also could underestimate the pooled prevalence of AUD.

## Conclusion

The results of the current review revealed that the prevalence of AUD among PLWHA was high when compared to the prevalence of AUD in the general population. The pooled prevalence of AUD was higher as it was measured by the AUDIT as compared to CIDI. We also found that the prevalence of AUD was higher in men than in women. The prevalence estimates of AUD showed significant variation across developed and developing countries. Screening and appropriate management of AUD among PLWHA is recommended. Further strong studies with representative sample size across the globe were warranted to strengthen our findings. Furthermore, further studies focusing on the magnitude of AUD across different countries as well as gender were warranted.

## Supplementary information

**Additional file 1.** Adapted NOS for Cross-sectional studies.

**Additional file 2.** Qualities of included studies in meta-analysis.

## Data Availability

All data generated or analyzed data in study are included in this article.

## Abbreviations

AUD: Alcohol use disorder
AUDIT: Alcohol used disorder identification test
CIDI: Composite International Diagnostic Interview
PLWHA: People living with HIV/AIDS
QOL: Quality of life
