# Supplementary figures and images for: The prevalence of alcohol use disorders among people living with HIV/AIDS: a systematic review and meta-analysis
Source: Subst Abuse Treat Prev Policy. 2019 Nov 14;14:52. doi: 10.1186/s13011-019-0240-3 (PMC6854786; doi:10.1186/s13011-019-0240-3)

**Additional file 1 : NOS adapted and modified for cross-sectional studies**


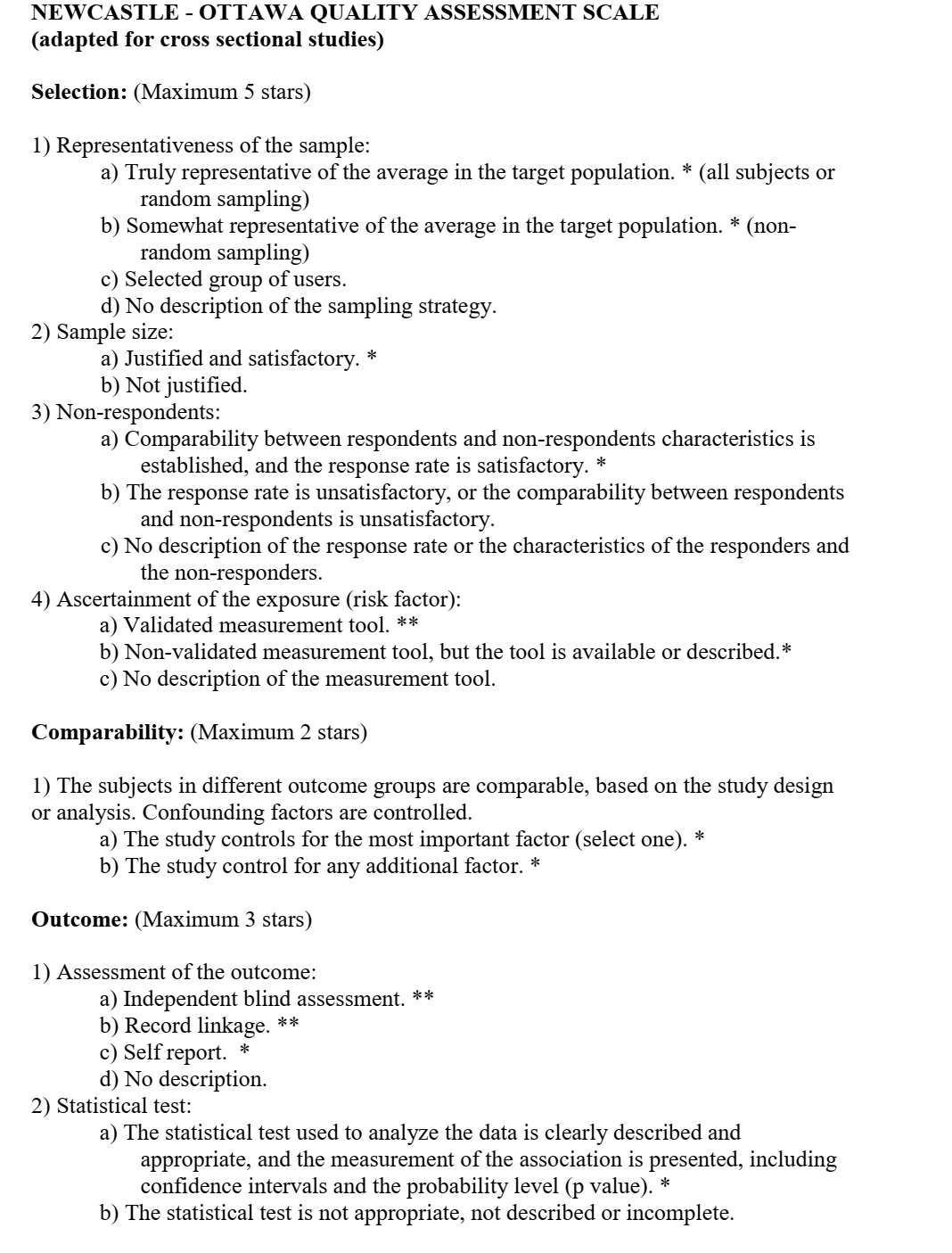

Supplement: Supplementary file 1 — Additional file 1. Adapted NOS for Cross-sectional studies. [file 13011_2019_240_MOESM1_ESM.docx]
