# Supplementary material for: The prevalence of alcohol use disorders among people living with HIV/AIDS: a systematic review and meta-analysis
Source: Subst Abuse Treat Prev Policy. 2019 Nov 14;14:52. doi: 10.1186/s13011-019-0240-3 (PMC6854786; doi:10.1186/s13011-019-0240-3)
Supplement: Supplementary file 2 — Additional file 2. Qualities of included studies in meta-analysis. [file 13011_2019_240_MOESM2_ESM.docx]

**Additional file 2:** The quality of studies included in final analysis

| Study name | NOS score | Quality level |
| --- | --- | --- |
| Silverberg et al., 2013 | 9 | High |
| Segni MT et al., 2017 | 8 | High |
| Silva et al., 2017 | 8 | High |
| Crane et al., 2017 | 9 | High |
| Nouaman MN et al., 2018 | 9 | High |
| Pokhrela et al, 2018 | 8 | High |
| Egbe et al., 2017 | 9 | High |
| Rosmary, 2015 | 5 | Low |
| Kibera A. et al., 2017 | 7 | Moderate |
| Goar et al., 2011 | 5 | Low |
| Mayston et al., 2015 | 9 | High |
| Zelalem B. et al., 2018 | 9 | High |
| Oliveira et al.,2015 | 6 | Moderate |
| Soboka et al., 2014 | 9 | High |
| Orwat et al., 2011 | 8 | High |
| Parsons et al.,2014 | 9 | High |
| D. Simon et al., 2014 | 8 | High |
| Medley et al., 2014 | 9 | High |
| Scott-Sheldon et al., 2014 | 9 | High |
| Jolley et al., 2016 | 9 | High |
| Idrisov et al., 2017 | 8 | High |
| Bultum J. A. et al., 2018 | 6 | Moderate |
| Wandera et al, 2015 | 8 | High |
| Farley et al, 2010 | 9 | High |
| Duko et al, 2019 | 9 | High |
